# Palliative care practices and policies in diverse socio-cultural contexts: aims and framework of the ERC globalizing palliative care comparative ethnographic study

**DOI:** 10.1177/26323524231198546

**Published:** 2023-09-11

**Authors:** Annemarie Samuels, Natashe Lemos Dekker

**Affiliations:** Institute of Cultural Anthropology and Development Sociology, Leiden University, Wassenaarseweg 52, Leiden, 2333 AK, The Netherlands; Institute of Cultural Anthropology and Development Sociology, Leiden University, Leiden, The Netherlands

**Keywords:** Brazil, culture, diversity, ethnography, globalization, good death, India, Indonesia, palliative care

## Abstract

**Background:**

Palliative care as a specialist professional practice of care for people with advanced illness is becoming increasingly influential worldwide. This process is affected by global health inequalities as well as cultural dimensions of approaching death and practicing care in life-limiting illness.

**Objectives:**

The European Research Council-funded Globalizing Palliative Care (ENDofLIFE) project aims to understand how palliative care policies, discourses and practices are translated, adapted and reconstituted in diverse socio-cultural settings and how cultural dimensions of approaching death and local practices of care shape palliative care implementation.

**Methods and Analysis:**

Using a multi-scalar and multi-sited ethnographic approach, the project uses person-centered ethnography, participant observation, semi-structured interviewing, focus group discussions and policy and discourse analysis at transnational, national and local levels. Ethnographic case-studies are conducted in Brazil, India and Indonesia.

**Discussion:**

The globalizing palliative care project develops a novel ethnographic methodology of studying *end-of-life care trajectories* through long-term participant observation with individual patients and families as they manage and practice formal and informal health care in advanced illness. By analyzing how patients and families experience and navigate care over time, complemented by stakeholder interviews, the study advances critical theoretical insight into the relation between (large-scale and dynamically traveling) palliative care models, policies and discourses on the one hand and the experience and practice of palliative care in the lives of patients and informal care givers in local health care practices on the other hand. Insights are expected to benefit culturally situated palliative care policies and practices.

## Background

Palliative care as a specialist professional practice of care for people with advanced illness is becoming increasingly influential worldwide. Its development in the 1960s and ’70s and subsequent spread around the world has been well documented.^1,2^ Over the last decade, there has been a substantial rise in health policy and scholarly attention for expansion of palliative care services to low- and middle-income countries.^3,4^ Emphasizing palliative care as a universal global health concern, the WHO recognizes palliative care as a human right.^5^ At the same time, anthropologists find that around the globe people have highly diverse ideas about good care, a good death and good dying,^6–8^ and not all cultural and religious contexts provide the same meaning to pain, or support disclosure of terminal illness to the patient.^9,10^ Moreover, showing that values of death and dying are continuously in flux and have been rapidly changing over the last decades, the Lancet Commission on The Value of Death has recently set out principles for ‘radically reimagining a better system for death and dying’.^11^

Given this current international attention to globalizing palliative care on the one hand and the cultural diversity of approaching the end of life on the other, it is important that we better understand how palliative care does or does not translate across diverse contexts. To address this question, the European Research Council (ERC)-funded project *Globalizing Palliative Care? A Multi-sited Ethnographic Study of Practices, Policies and Discourses of Care at the End of Life* studies how palliative care policies, discourses and practices are translated, adapted and reconstituted in diverse socio-cultural settings. Approaching the globalization of palliative care as a multi-scalar process, it also investigates how other, non-specialist and culturally diverse practices of end-of-life care impact palliative care practices. As elaborated below, it does so through in-depth ethnographic case studies in Brazil, India and Indonesia.

### Global inequalities in palliative care provision

Extensive inequalities in palliative care provisions persist between high-income countries and low- and middle-income countries (LMICs).^3,12,13^ While many LMICs have developed palliative care provisions in the last decades, and there are several local success stories [e.g. ref. ^14^], often such provisions remain limited to major cities and national hospitals, and are not integrated with the entire national health care system.^3,4^ Despite significant dilemmas of justice in resource allocation^15,16^ this is not merely an economic problem: many countries face strict policies on opioid provision, a lack of palliative care training facilities, and cultural taboos on discussing death and dying.^17–21^

### Cultural factors influencing palliative care implementation

An important cultural factor influencing palliative care implementation is that in diverse socio-cultural settings, discussing death and dying with the patient may be considered problematic or inappropriate.^9,22^ Not disclosing to patients that their illness is terminal may be seen as an act of care.^8,10,23^ Cultural and religious values influence people’s opinions on whether the hospital, the hospice or the home is seen as the best place to die.^24^ In addition, religious perspectives inform discussions on the appropriateness of pain medication.^25,26^ More generally, suffering and pain are experienced, and valued, in diverse ways both within and across societies.^27,28^

### Cultures of palliative care

While such cultural and religious dimensions are sometimes described as barriers to the implementation of palliative care, this project investigates how palliative care may be *differently* translated to diverse national and cultural settings and how non-specialist forms of care at the end of life shape this process. We have adopted this approach, since it allows for the multiplicity of moral perspectives on good care and a good death and takes into account that transnational policies and discourses of palliative care are themselves culturally constructed and continuously developing. We thereby contribute to addressing a growing demand for a more nuanced view of both the cultures of palliative care provision and the cultural diversity of end-of-life care practices around the world.^29–31^

## Aims and objectives

This article aims to highlight the key objectives, methods and expected scientific contributions of the globalizing palliative care research project, which is at the time of writing in the data collection and analysis phase.

The two central objectives of the project are:

1. To understand how palliative care policies, discourses and practices are translated, adapted and reconstituted in diverse socio-cultural settings.
2. To gain in-depth insight into the ways in which culturally diverse, non-institutional practices of end-of-life care impact palliative care practices, policies and discourses.

The overarching scientific aim of the project is to theorize what is universally shared and what is culturally specific in end-of-life care. End-of-life care is thereby broadly defined as including specialist palliative care (provided by health care workers and others with palliative care training) as well as all other non-specialist practices of care at the end of life, including informal and family care.

## Method

### Study design

*The Globalizing Palliative* project uses a multi-scalar and multi-sited ethnographic comparative approach. The project is multi-scalar, as it studies the dynamics of palliative care practices, policies and discourses between international organizations, national-level institutions and local care settings. It is multi-sited, because it employs a comparative case-study method to examine these dynamics in three countries with emerging palliative care services: Brazil, India, and Indonesia.

At the transnational level, the project looks at the articulation and global mobility of palliative care discourses, policies and practices. At the national level, the project studies institutional care assemblages of policy, health care systems and activism. At the local level, the project examines what we define as *care trajectories*. This entails long-term ethnographically following patients with advanced illness and their immediate caretakers through various forms of end-of-life care over time. The methodological tool of following care trajectories will be further elaborated below in the section ‘scientific contribution’. In the overarching analysis, the project compares the case studies and examines the dynamics between the international, national and local levels.

### Country case studies

In-depth ethnographic case studies are conducted in three countries: Brazil, India and Indonesia. These countries have been selected for several reasons. First, all three have *emerging palliative care contexts*, as all are identified as category 3 (India and Indonesia 3a and Brazil 3b) on the four-point scale of palliative care provision of the Global Atlas of Palliative Care.^4^ This means they all have some palliative care services but these are not well integrated into the mainstream health care system.

In Brazil, palliative care has been developed since the 1980s, a national association of palliative care has been founded in 1997, and there have been a number of government initiatives for increasing its availability.^32^ Yet palliative care is not well integrated in the public healthcare system, large differences in access exist across the country, and local unstructured initiatives remain an important source of service.^33,34^ In India, palliative care services were developed for over three decades. Coverage remains very limited^19,35,36^ with the exception of the state of Kerala, where community initiatives in palliative care are successful in generalized provision.^14^ Indian palliative care organizations and physicians have over the last decade made major contributions to further education and development of palliative care services.^37^ Finally, in Indonesia, palliative care has been developed since 1992. While services are as yet limited to tertiary health facilities and limited non-governmental initiatives in a few major cities,^20,38,39^ a growing number of policy, community and scholarly initiatives is currently being developed to implement advance care planning.^40^

In all the three countries, important reasons for the limited availability of palliative care have been noted to be the insufficient medical specialization options, insufficient policy attention, limited availability of opioid analgesics and cultures of non-disclosure between physician and patient – a practice conflicting with prevailing global palliative care models as developed through the modern hospice movement.^20,32,35,41^ The selected countries are further comparable because they are democratic countries, with rising middle classes, expanding availability of biomedical treatment and aging populations. Moreover, they are also complementary because of different socio-political systems and dominant religions – Christianity, Hinduism, and Islam respectively – as well as different health insurance systems.

### Methodology

The project draws on qualitative research methods. Data are gathered primarily through ethnographic fieldwork, which offers detailed and comprehensive insight into subtle and sensitive dimensions of life, as it entails that researchers immerse themselves in the daily lives and practices of their research subjects.^42^ Key qualitative methods within the ethnographic toolkit employed by this project are:

- person-centered ethnography (which mean long-term participant observation and recurrent interviewing) with patients and families;
- participant observation (in care trajectories and caregiving activities, policy meetings, and medical consultations);
- in-depth and semi-structured interviews (with officials, policy-makers, patients, relatives, medical staff and activists);
- focus group discussions (with policy-makers, medical doctors and nurses); and
- policy and discourse analysis.

Ethnographic methods are key to this project because they grant privileged access to each of the relevant levels of analysis. First, to understand care trajectories, we must study particular patients and families over time in their everyday contexts. Given the sensitivity of the topic, this long-term engagement allows the researcher to build rapport and facilitates observing and asking questions about caregiving practices, attitudes toward death and dying, and access to different forms of health care. The researchers actively seek to include participants from diverse identities and backgrounds with respect to gender, ethnicity, class and religion. Children are not enrolled as research participants, either as patients or family caregivers. However, adult participants’ discussion of their role, presence or care contribution will be included.

Second, ethnographic methods allow us to trace the compositions and transformations of institutional care assemblages, which consist of networks of people, discourses and practices, over time. Through participant observation in networks of medical professionals, policy makers, and insurance companies as well as recurrent interviews and focus groups with stakeholders, institutional care assemblages can best be identified, described and analysed. Third, palliative care discourses and policies travel around the globe through concrete professional training, policy modules and exchange of medical expertise. Understanding how these discourses and policies get translated, mediated and re-constituted in the process requires an ethnographic study among policymakers, practitioners and activists.

Data will be analysed and compared using a grounded theory approach and principles of thematic analysis for qualitative data.^43^

## Discussion: scientific contribution

This section discusses key scientific contributions of the project. We first discuss the methodological contribution of following care trajectories at the end of life that is developed through this project and the insights that developing and implementing this methodology has gained so far. Secondly, we show how data gathered through this methodological approach allow for critical theoretical insight into the relation between (large-scale and dynamically traveling) palliative care models, policies and discourses on the one hand and the experience of these in the lives of patients and informal care givers in local health care practices on the other hand. As such, we suggest, an in-depth focus on the experiential trajectories of care of patients and informal caregivers reveals socio-cultural dimensions of palliative care and the ways in which global and national developments are negotiated, situated and adapted in local practices. This leads us to theorizing how the global and local intersect in ‘global’ palliative care. Finally, we discuss the expected impact of this project, which can be found in its novel analytical framework of studying the intersection between globally traveling palliative care models and local culturally diverse end-of-life care practices, the advancement of development of culturally sensitive palliative care models, and the methodological tool of ethnographically studying *end-of-life care trajectories*.

### Studying care trajectories: developing new methodologies

A key contribution of this project is the development of a methodological approach to studying ‘care trajectories’ at the end of life. The ethnographic approach taken in this project, involves a commitment to long-term and in-depth engagement with the research sites and participants. The researchers build relationships of trust with research participants and participate and observe in their everyday lives.

This allows us to ‘follow’ the care trajectories of particular patients as they go about seeking treatment and decision-making in relation with relatives, caregivers and medical professionals. Our approach focuses on the one hand on the experiences of patients, families and caregivers, and the narratives they construct to provide these experiences with meaning. On the other hand, we focus on how people actively give shape to their care trajectories by engaging in particular care relations and by navigating formal and informal care structures. Indeed, preliminary results from the case studies show that care trajectories often involve a continuous crossing of institutional and non-institutional boundaries and specialist and non-specialist forms of care.

Following care trajectories thus involves not only institutional care. Rather, the focus on how people navigate and give shape to different care systems enables a more complete view of how care is provided at the end of life and how care needs change over time, and it allows us to understand the barriers for people to access specialist care. In other words, key to understanding care trajectories, is looking at the variety of aspects and relations of care that come to matter to the patient. To do so, researchers need to have access to the informal sphere of family caregiving and everyday care in people’s homes. It also implies involving not only the patient themselves, but a variety of others who may be regularly or sporadically involved in care. This multiplicity of perspectives is considered part and parcel of the ethnographic research methodology.

Hence, the methodological approach of ethnographically following care trajectories results in an encompassing view of often complex care situations and their development over time. The long-term and in-depth engagement of this methodological approach can bring into view how patients, kin and care professionals speak about illnesses, and perhaps even more importantly, is the only way to ‘see’ *silences*, aspects of the care trajectories that are not openly discussed. For example, after getting to know a patient and family well, the ethnographer who observes a patient’s consultation with their physician, may notice what parts of the patient’s experience the patient/family highlights in the conversation and what parts they do not talk about. Such silences are impossible to gather from interviews or surveys, and only become apparent and explainable through long-term engagement with individual patients and their caregivers. Ultimately, therefore, the ethnographic approach of following care trajectories provides an in-depth understanding of the cultural context within which end-of-life care takes place, and more particularly, of how the decisions and practices that take place in these trajectories acquire meaning in relation to their social and cultural setting.

A key strength of this approach is thus that it enables us to study end-of-life care practices up-close. The researchers are spending valuable time with patients and families, come to understand many aspects of their lives, are present when sensitive personal information is discussed, such as diagnosis or prognosis, and may participate in exceptional moments that are usually only witnessed by close family and medical professionals, including the moment of death. This type of research with people in vulnerable situations and the proximity between the researcher and the research participants, requires a continuous reflection on the researcher’s position, their own emotions and research ethics. Moreover, as the researcher follows care trajectories, this approach requires the researcher to loosen their control over the research and allow themes that are of importance to research participants to emerge as their situation changes. To allow for these dimensions to be fully integrated in the approach, the globalizing palliative care project has organized reflective meetings and workshops on ethics and emotions of ethnographic fieldwork in end-of-life care early on in the project and establishes regular reflection on emotions, ethics and emergent themes throughout data gathering and analysis.

### Developing palliative care theory in global perspective

As discussed above, the long-term ethnographic research of this project is key to understanding how patients and informal caregivers *navigate* and *experience* end-of-life care trajectories, including those dimensions that remain relatively muted. In-depth explorations of individuals’ and families’ ways of navigating care allows for a bottom-up perspective on the social structures that shape end-of-life experiences. This methodological perspective builds on, and further advances, theoretical work on death and dying as a social and cultural process. The following two paragraphs highlight two examples of this theoretical work, namely the pathbreaking studies by Sharon Kaufman and Scott Stonington, conducted in the United States and Thailand respectively.

In a highly influential ethnographic study of dying in American hospitals, Sharon Kaufman^44^ found that patients and families were often confused about the stage of illness they were in and whether they were waiting for death or for recovery. Hospital and insurance structures made it important for physicians to move the patient to the next stage of (palliative) treatment or home care. Yet, often their efforts to ‘move the patient along’ to this next stage did not match patients’ and family’s expectations. Kaufman’s in-depth engagement with patients’ and physicians’ often frustrating experiences revealed the force of cultural understandings and expectations of ongoing treatment for cure in the American hospital context in a time of increasingly critical discussions about futile and invasive treatment at the end of life.

In a rather different setting, Scott Stonington^8^ has shown how in Thailand, cultural values of kinship obligation shape family’s efforts to pursue curative treatment until the patient is very close to dying. While patients may individually not feel the need to keep pursuing treatment, and might rather be inclined to opt for palliative care, they at the same time often do not voice such individual opinions. The reason they keep quiet about preferences to not prolong treatment is to not take away from their family members valuable opportunities to gain karmic merit by providing what they consider to be the best possible care. Such insight in (silent) cultural dimensions of practices of care at the end of life, Stonington shows, are vital to understanding how the modern palliative care movement is at once embraced in and adapted to the Thai context.

Around the world, anthropologists show a diversity of approaches to what constitutes a ‘good’ death [e.g. refs. ^45,46^] and reveal how what people value as ‘good’ care is locally situated and shaped by political and class relations [e.g. refs. ^47,48^]. Therefore, as the case studies by Kaufman and Stonington show, professional palliative care practice in diverse contexts and places may assume diverse forms. The globalizing palliative care project studies how the individual care trajectories of patients and caregivers reveal how, for example, discursive models of diagnosis disclosure, but also ways in which palliative care is or is not included in health coverage plans, shape the local configuration of palliative care models. On a global level, the project asks what elements in this worldwide amalgam of practices of care ensure that these are called ‘palliative care’? In other words: What are shared dimensions in globalizing palliative care? How do they relate to the local diversity in end-of-life care practices? Our initial findings from exploratory field visits and literature study point at the dynamic and creative ways in which the concept of palliative care is invoked (or, in sensitive contexts, downplayed), the various ways in which professionalization of end-of-life care is imagined and the diversity of values – including those shaped by Cicely Saunders’ work and legacy – that shape the practices of what local health care workers or institutions themselves call ‘palliative care’.

Next to empirically teasing out the varieties in local palliative care practices, one major theoretical scientific contribution that this project aims to make through the analysis of the data, then, is to conceptualize how the ‘global’ in global palliative care is made and what this means to the dynamic development of the field of palliative care at large. To do so, it is crucial to realize that globally circulating models and discourses are themselves always culturally shaped and in flux.^49^ In order to make this conceptual step, the objectives of the project as highlighted in this article are to understand the process of ‘globalizing palliative care’ at the intersection of (1) globally/internationally circulating policies, discourses and practices that are translated, adapted and reconstituted in diverse socio-cultural settings; and (2) the ways in which culturally diverse, non-institutional practices of end-of-life care impact palliative care practices, policies and discourses. To put it differently, studying the connections between traveling (cultural) models of palliative care and local innovations and practices that are currently developed and may (later) travel on to other places, we theorize how global palliative care is locally made.

### Impact

The impact of the project is expected to be threefold:

1. The project offers a high level of empirical detail on end-of-life care that helps adjusting the geographical imbalance in studies of palliative care.^50^ It moreover develops an analytical framework for understanding the global mobility of palliative care models in relation to culturally diverse local end-of-life care practices. This framework can be used in further analyzing and theorizing universally shared and locally specific dimensions of end-of-life care.
2. Insights from this project will benefit the development of culturally situated palliative care policies and practices. The need for developing such culturally sensitive models is becoming an increasingly important topic in the field of palliative care.^30,51^
3. The project develops a methodological ethnographic tool of *end-of-life care trajectories*, which will be widely applicable in the longitudinal study of care in terminal and advanced illness.

## Conclusion

As palliative care services are implemented in an increasingly diverse range of socio-cultural settings around the world, it is essential that we better understand how this process works and how it is impacted by local practices of care at the end of life. The ERC-funded Globalizing Palliative Care (ENDofLIFE) project builds a multi-scalar comparative ethnographic approach and develops a novel methodological tool of following *end-of-life care trajectories* in order to address this pressing issue. The project thereby contributes to methodological innovation, theorization of globalizing palliative care and practical development of culturally situated palliative care policies and practices.

## Declarations

**Ethics approval and consent to participate:** The study has been approved by the European Research Council Executive Agency, conditional on ethics requirements to be met during the course of the project (851437_ENDofLIFE_EthSR) and the Ethics Review Committee of the Social Sciences of the Faculty of Social and Behavioural Sciences at Leiden University (Unnumbered, approval date 20 July 2020).

**Consent for publication:** Not applicable.

**Availability of data and materials:** Not applicable.
